# Recognition of patients with medically unexplained physical symptoms by family physicians: results of a focus group study

**DOI:** 10.1186/s12875-016-0451-x

**Published:** 2016-05-12

**Authors:** Madelon den Boeft, Danielle Huisman, Johannes C. van der Wouden, Mattijs E. Numans, Henriette E. van der Horst, Peter L. Lucassen, Tim C. olde Hartman

**Affiliations:** Department of General Practice and Elderly Care Medicine, EMGO Institute for Health and Care Research, VU University Medical Center, Van der Boechorststraat 7, room D5.40. 1081 BT, Amsterdam, The Netherlands; Department of Public Health and Primary Care, Leiden University Medical Center, Albinusdreef 2, 2333 ZA, Leiden, The Netherlands; Department of Primary and Community Care, Radboud University Nijmegen Medical Center, Nijmegen, The Netherlands

**Keywords:** Medically unexplained physical symptoms, Recognition, Diagnostics, Family medicine, Patient profiles, Somatisation

## Abstract

**Background:**

Patients with medically unexplained physical symptoms (MUPS) form a heterogeneous group and frequently attend their family physician (FP). Little is known about how FPs recognize MUPS in their patients. We conducted a focus group study to explore how FPs recognize MUPS and whether they recognize specific subgroups of patients with MUPS. Targeting such subgroups might improve treatment outcomes.

**Methods:**

Six focus groups were conducted with in total 29 Dutch FPs. Two researchers independently analysed the data applying the principles of constant comparative analysis in order to detect characteristics to recognize MUPS and to synthesize subgroups.

**Results:**

FPs take into account various characteristics when recognizing MUPS in their patients. More objective characteristics were multiple MUPS, frequent and long consultations and many referrals. Subjective characteristics were negative feelings towards patients and the feeling that the FP cannot make sense of the patient’s story. Experience of the FP, affinity with MUPS, consultation skills, knowledge of the patient’s context and the doctor-patient relationship seemed to influence how and to what extent these characteristics play a role. Based on the perceptions of the FPs we were able to distinguish five subgroups of patients according to FPs: 1) the anxious MUPS patient, 2) the unhappy MUPS patient, 3) the passive MUPS patient, 4) the distressed MUPS patient, and 5) the puzzled MUPS patient. These subgroups were not mutually exclusive, but were based on how explicit and predominant certain characteristics were perceived by FPs.

**Conclusions:**

FPs believe that they can properly identify MUPS in their patients during consultations and five distinct subgroups of patients could be distinguished. If these subgroups can be confirmed in further research, personalized treatment strategies can be developed and tested for their effectiveness.

## Background

Medically unexplained physical symptoms (MUPS), physical symptoms for which no adequate medical explanation can be found after a proper examination, are common in primary care and may have a major impact on the daily life of patients [1–3]. We know that patients with MUPS constitute a heterogeneous group. This heterogeneity is due to a broad range of clinical symptoms [4], variety in sociodemographic characteristics such as age, employment status and educational level, and lastly to psychiatric comorbidity [5]. Almost all kinds of MUPS can be presented to FPs in varying degrees of severity. Functional somatic syndromes such as fibromyalgia (FM), irritable bowel syndrome (IBS) and chronic fatigue syndrome (CFS) are also referred to as MUPS.

Currently, few effective interventions for MUPS are available. Up to now, only cognitive behavioral therapy (CBT) has been shown to have a small benefit by reducing symptoms and functional impairments [6]. The varying and disappointing treatment outcomes can be due to this heterogeneity, as different subgroups of patients may have different needs and may benefit from personalized and targeted health care. In previous studies among patients with FM the authors identified two subgroups, patients with pain avoidance and patients with pain persistence, and these subgroups benefitted from a different treatment approach [7–9]. Also, several studies highlighted the relevance of the heterogeneity among patients with CFS for their treatment response and the need to explore this heterogeneity more in to depth [10, 11]. In line with these studies and in the light of the scarcity of effective treatments, identifying distinct subgroups of patients with MUPS might be a way forward to develop more targeted interventions. Even though patients with MUPS are frequently seen by FPs, little is known about the actual process of recognizing MUPS by FPs. With this in mind, we conducted a focus group study that specifically addressed the following two research questions: 1) How do FPs recognize MUPS in their patients and 2) Which distinct subgroups of patients with MUPS do FPs recognize?

## Methods

### Design, setting and participants

We chose the focus group method because group dialogues tend to generate rich information as the input of any participant may trigger other participants to share their experiences and thoughts in a natural and dynamic way [12].

We started with analysing three focus groups discussions with FPs from a study previously conducted by olde Hartman et al. [13]. In these focus groups many aspects concerning the recognition of MUPS by FPs and delineation of MUPS were addressed. Therefore we chose to analyse their first three focus groups before initiating new focus groups. Thirteen FPs altogether participated in these first three focus groups and each session lasted approximately 1 h and a half. Detailed information is described elsewhere [13]. After analysing these focus groups, we organized three additional focus groups with FPs to discuss the recognition of MUPS in more depth and to discuss the existence of possible subgroups of MUPS.

For the recruitment of participants, we consulted the staff members of the department of general practice and elderly care medicine of the VU University medical center (VUmc) Amsterdam for names of FPs who might be interested in participating. We invited the FPs by email, letter and/or phone. Similar to olde Hartman et al, we used a purposive sampling strategy with the aim to increase the external validity of the results. FPs were sampled aiming at variation on the following characteristics: age, gender, working experience, geographic location of practice, academic working career versus non-academic career and affinity with MUPS versus no special affinity with MUPS. Given the qualitative approach, the purposive sampling strategy and the relatively small sample size, we did not take into account the FP characteristics in the analysis.

We invited 52 FPs of whom 32 were willing to participate and 16 actually participated in one of the three focus group discussion. The other 16 FPs were not able to participate due to logistical reasons. Each focus group included four to eight FPs and the sessions lasted approximately 1 h and a half. Table 1 summarizes the information of the participants of the six focus group discussions included in our study.

**Table 1:** Participant information of all six focus groups

	Number of FPs (*n* = 29)
Gender	
Male	16
Female	13
Age in years (range)	51 (31–67)
Experience as a FP in years (range)	19 (0–34)
Working hours^a^	
Full time	15
Part time	13
Not practicing at the moment	1
Type of practice	
Solo	1
Pair	8
Group	16
Self-employed^b^	3
Not practicing at the moment	1
Urbanization	
Rural	14
Urban	13
Variable^b^	1
Not practicing at the moment	1

*FP* family physician. ^a^Full time means 80–100 % working; Part time means less than 80 % working. ^b^Self-employed FPs work in different family practices

An independent, skilled moderator without any interest in the outcome facilitated the discussions and made sure all themes from the interview guide (Table 2) were discussed. After each focus group discussion, we adapted and refined the interview guide, allowing new ideas and thoughts that emerged in earlier stages of the analysis to be brought forward in subsequent sessions. All discussions were transcribed verbatim according to a transcription guideline and entered in the qualitative software program Atlas.ti version 7.

**Table 2:** Interview guide

1	Think of a patient with MUPS
	a. What are characteristics of this patient?
	b. What are characteristics of the patient’s complaints?
2	Regarding recognizing MUPS in your patients:
	a. Many doctors say that they know whether they are dealing with a patient with MUPS within a short time. What is your opinion and experience regarding this issue?
	b. Some of the complaints that you almost instantly consider to be MUPS are indeed MUPS and some are not. When do you adjust your hypothesis?
	c. Do hunches play a role in the recognition of MUPS? Or feelings that are evoked in you? If so, can you describe these hunches and feelings?
	d. Does the background of the patient (or the story the patient tells with regard to his complaints) play a role in the recognition of MUPS? How and to what extent?
	e. Does recognition depend on how much you can empathize with the patient or the complaint? Do you still consider it MUPS when you empathize?
	f. Does the patient’s insight in social or psychological contributors to his complaints play a role in the recognition of MUPS? How and to what extent?
	g. Does the quality of the physician-patient relationship play a role in the recognition of MUPS? How and to what extent?
	h. Do you still consider it to be MUPS when a patient is agreeable and you like him?
3	I would like to hear your opinion on the following statement: "Every doctor has his own type of MUPS patient". (Does the personality of the doctor influence the recognition of MUPS?)
4	Is there a difference, with regard to the recognition of MUPS, between patients who you have known for long time and patients you hardly know?
5	Are you able to distinguish different subgroups of patients with MUPS? How?

### Ethics

The medical ethics committee VUmc approved the study (reference number 2015.216). All FPs provided written informed consent to participate, for the usage of the data and to publish this manuscript.

### Analysis

We applied the principles of constant thematic comparative analysis [14]. First, two of the authors (MdB, DH) carefully read and familiarized themselves with the transcripts. After that, they independently coded the transcripts and categorized the codes by theme to explore similarities and differences between responses of the FPs regarding the recognition of MUPS and subgroups of patients. While analysing, the researchers had the Dutch College of General Practitioners MUPS guideline in mind [15]. This guideline uses a framework that covers specific dimensions of the complaint(s) and general MUPS characteristics and pays attention to the doctor-patient relationship. The complaint dimensions (i.e. the somatic, cognitive, emotional, social and behavioural dimension) are rooted in the biopsychosocial model [16]. The biopsychosocial model assumes that perceived health is associated with all the dimensions of human existence and that every human being is in constant interaction with their environment.

All themes were discussed by MdB and DH and refined after each focus group analysis. Disagreements and doubts were frequently discussed with two senior researchers (PL, ToH).

The construction of distinct subgroups of MUPS patients was based on responses and perceptions of the FPs in two consecutive steps: 1) analysis of the direct responses of FPs on the question if they could recognize or distinguish distinct subgroups and more specifically, in what way; and 2) analysis of the responses of FPs that emerged spontaneously during the discussions of other topics and seemed to co-occur or combine into a pattern relating to the recognition of subgroups. The subgroups were organised by describing their most explicit and/or predominant characteristic and the behaviour of the patient during the consultation as reported by the FPs, including their feelings towards these subgroups that were used as signifiers for recognition.

To internally validate our findings we performed a member check among all participating FPs and five FPs who are staff members of VUmc who had not participated in any of the focus groups. In this member check we presented the results of our study and asked the FPs to determine if these were consistent with their perceptions and/or experience. After the sixth focus group we concluded that saturation was reached because no new themes emerged from the transcripts of this focus group study.

## Results

### How do FPs recognize MUPS in their patients

All FPs were familiar with the phenomenon of MUPS and thought about patients with MUPS as a heterogeneous group. Most FPs believe that they can easily distinguish between explained and unexplained symptoms when they were familiar with the patients’ medical background and context. They stated that they needed follow-up consultations to diagnose MUPS in patients whom they did not know well. Several FPs described that the first signal is often that what patients are telling about their symptoms does not make sense to them.

FPs reported taking diverse characteristics, both objective and subjective, into account when considering MUPS as their working hypothesis. More or less objective characteristics are multiple, non-specific symptoms that remain undiagnosed, frequent and long consultations and a relatively high number of referrals for additional diagnostics or to specialists. Amongst subjective characteristics are the feelings, often negative, that MUPS patients evoke in FPs, like irritation and resistance. Another indication of MUPS seems to be that during the consultation, FPs feel forced to repeatedly switch between the diagnostic and management phase, as the patient does not agree with the actions proposed by the FP. This may result in a struggle. Both the switching between phases and the struggle can make FPs aware that they are dealing with MUPS. Quotes on the recognition of MUPS are summarized in Table 3.

**Table 3:** Quotes of FPs in relation to the recognition of MUPS

FG5;FP5: ‘I believe I know what is going on within 30 s, like many of us. When I think within 2 min “I do not have a clue of what is going on here”, then I start to think “This can be MUPS”.’
FG5;FP3: ‘Well, we all know the consultation where things go as you have planned. You do what you always do, start with taking history, then physical examination, then you often have a diagnosis and then you discuss the strategy. But with MUPS patients, what I usually notice is that the discussion does not go so well and you switch between phases. And you think, what is going on? That is a first possible recognition clue.’
FG2;FP1: ‘When someone consults me with chest pain during exercise that disappears after 2 min at rest, that is something completely different from when they present many complaints and we often call them atypical, right? It does not fit with a specific disease. They have a headache, but when you talk about the headache they also have back pain and when you are finished with the back pain, they also feel tingles and with everything together, it just does not make sense.’
FG4;FP5: ‘A long list of episodes.’ FP1 and FP4: ‘Yes.’ FP3: ‘A long history…’ FP5:’…Without any serious diseases.’ FP2: ‘With many referrals for additional examinations or to specialists.’ FP1: ‘They remain at the complaint level, like headache or stomach ache or fatigue or dizziness.’
FG1;FP1: ‘I often use it as a diagnostic tool for MUPS, that I get irritated by patients.’
FG4;FP1: ‘What I notice is that many doctors have the same basic feeling about these patients and how they recognize them: the exhaustion, the desperation of the doctor and the way they easily get into a fight with these patients.’

*FG* focus group, *FP* family physician. The numbers correspond with the focus groups session and the family physician

The process of recognizing MUPS is not straightforward. Most FPs describe that they recognize MUPS based on subtle feelings. The degree to which FPs are able to empathize with their patients can strengthen them in their idea that symptoms might be unexplained. Most FPs stated that when they did not feel empathy for their patients, they were more often inclined to recognize symptoms as MUPS. Therefore the lack of empathy is often used as a signifier for recognition. The degree of empathy is influenced by mainly two factors. First, FPs reported that when they liked their patient, they could better empathize. Second, when FPs were familiar with the patient’s context and when the patient could verbalize their symptoms and relate their symptoms to their context, FPs were also more able to understand and empathize (e.g. low back pain due to heavy physical work or being tired when having a lot of stress makes sense). However, the presence of empathy does not completely rule out the possibility MUPS. FPs may empathise and still consider MUPS.

Finally, the personality of the FP, their affinity with MUPS, the years of experience and consultation skills were reported to play an important role in the process of recognition. More work and life experience and better skills in coping with MUPS made recognition easier. Quotes on the contributing factors in recognizing MUPS are summarized in Table 4.

**Table 4:** Quotes of FPS in relation to the contributing factors in the recognition of MUPS

FG3;FP2: ‘For FPs it is important to know the context of a patient. If you know about the busy shoe store, then you can empathise more with MUPS.’
FG4; FP1: ‘So if you know more about the context, you can better empathise.’ FP5: Well, it causes less irritation FP1: When you have more experience or when you have a longer relationship, you just have more information and you recognize it sooner.
FG6;FP2: ‘Some patients with a certain personality structure; it is possible that you are just a bit more sensitive to them. So with some patients you will sooner consider “Could this be MUPS?”’

*FG* focus group, *FP* family physician. The numbers correspond with the focus groups session and the family physician

### Which distinct subgroups of patients with MUPS do FPs recognize?

Most FPs could not directly describe distinct subgroups when asked during the last three focus group discussions. However, apart from this specific question, the subject of distinct subgroups and specific characteristics of patients came up several times. From these characteristics five distinct subgroups emerged from the focus group data. These subgroups were not mutually exclusive, as patients fulfilling the criteria for one subgroup could also have characteristics belonging to another subgroup (e.g. patients with feelings of anxiety may also have a low mood or can be distressed and these symptoms can be inter-related as well). We discerned the following five subgroups of patients according to FPs: 1) the anxious MUPS patient, 2) the unhappy MUPS patient, 3) the passive MUPS patient, 4) the distressed MUPS patient, and 5) the puzzled MUPS patient. Below we describe each subgroup including (a) the explicit and/or predominant characteristics and the behaviour of the patient during consultations as perceived by the FP and (b) subjective feelings towards the patients in these subgroups that can be signifiers for recognition. The quotes regarding the subgroups are summarized in Table 5.

**Table 5:** Quotes of FPs in relation to the different subgroups of patients with MUPS

The anxious MUPS patient	*FG4;FP4: ‘I often see anxious patients, who have a bad connection with their body and who are in panic because of it and are not easily reassured. They always hope to find reassurance in all kinds of additional examinations.’* *FP1: ‘Yes’. FP2: agrees*
	*FG5;FP2: ‘Is it not predominantly anxiety or an alarming feeling. It is constantly being overwhelmed by signals, physical signals that they cannot make sense of. I believe that patients are being overwhelmed and do not know what to do with all those symptoms. And therefore they come directly to us, as an authority to tell them what it is.’*
	*FG5;FP3: ‘I am sure that they feel the tingles, the palpitations and the headaches and then they think: “Oh My God, what is this? Let this be nothing serious.” They tend to give a catastrophizing explanation to it.’*
The unhappy MUPS patient	*FG1;FP3: ‘It is the recurrent thing, I mean, the fact that patients come back all the time with headache and then stomach ache and then the next time something else again, while their mood clearly fluctuates, than you do not have the depressive disorder. So it is the time that clarifies it.’*
	*FG2;FP1: I think that in patients with MUPS their mood is not as severely disturbed as in the case of a real depressive disorder. But in some patients with MUPS their mood can be low due to their symptoms. In the consultation room they can be apathetic.’*
The passive MUPS patient	*FG4;FP4:‘They hand the problem over to you and you should have the solution. They want to take tablets, but really working on solving their symptoms, they do not want that. They want it all, but not coming from them.’*
	*FG2;FP3: ‘They do not have the coping strategies to get over it. They are powerless.’* *FP1: ‘Yes, it just happens to them.’* *FP3: ‘It just happens to them and they cannot defend themselves.’* *FP2: ‘Yes, that is it. It happens to them and they express it in a certain manner: with a stomach ache at that certain moment.’*
	*FG1;FP4: ‘There is a group that always externalizes problems and symptoms. This can evoke a feeling of irritability in the doctor. It makes me feel powerless, because I do not get a way in.’*
	*FG5;FP5: ‘They look for an explanation, but an external one, not in themselves.’*
The distressed MUPS patient	*FG4;FP1: ‘Patients with moderately severe MUPS are often people who have periods where they are just not so comfortable, because, I do not know, they have troubles at work or in their relationship. Everyone has phases in their lives when they are feeling suboptimal. These [patients] are the easy ones’.*
	*FG4;FP3: ‘A high stress-level and a high level of expectations of themselves. So with a certain group of patients I often think about a burnout. If you burden yourself long enough, you will eventually get MUPS.’*
	*FG6; FP4: There are patients where you do not know what is wrong and where you think “Maybe there is some kind of abnormality t5:39 that we just have not discovered yet”.’* *FG4;FP5: ‘Patients with a different ethnic background have a tough life and when they consult with symptoms from the musculoskeletal system I think “Yes I understand those symptoms, I would have had the same symptoms with that kind of work”.’*
	*FG3;FP2: ‘I think that they sometimes persist in their own model of explanations; they do not want to look in other directions. There are so many psychosocial problems. People do not make choices or they are completely overloaded and then I think “Yes, with three children and this and that, I would be very tired”, but they seem to believe that everything should be possible or something like that.’*
	*FG5;FP1: ‘Sometimes you just have vulnerable patients, who do not have a strong support system and therefore they come to you.’*
The puzzling MUPS patient	*FG1;FP1: ‘We all know people who present themselves extremely balanced in your consultation room and tell you very clearly that they have symptoms and in whom you find zero abnormality. Nothing wrong at home, or something like that. Absolutely no abnormality at all.’*
	*FG1;FP3: ‘Of course there are some patients where there is absolutely no explanation at all and I am more inclined to keep searching for one and to refer them for additional examinations.’*
	*FG6; FP4: There are patients where you do not know what is wrong and where you think “Maybe there is some kind of abnormality that we just have not discovered yet”.’* *FP2: ‘I agree that is possible.’* *FP4: ‘Yes, that we cannot give an explanation with our current knowledge but in 24 years our diagnosis may be totally different.’* *FP3:’There are certain things still unexplainable now, but maybe not in another 100 years. Lyme is always a good example of such a thing.’*
	*FG6;FP1: Often they have given you cues in the history taking phase. So you can use physiology and certain explanation models, like adrenaline that is released in a certain situation, which can give you palpitations. But when there is absolutely no clue, then I think “This is really unexplained”.’*

*FG* focus group, *FP* family physician. The numbers correspond with the focus groups session and the family physician

### The anxious MUPS patient

According to FPs some patients specifically focus on bodily signals, tend to misinterpret harmless signals as disturbing and alarming and become anxious. Most FPs think that these anxious MUPS patients have an abnormal body experience. Anxiety may also be present in a more generalized way, where patients also worry about other aspects of life.

FPs reported that during consultations, patients tend to express disproportional worry and FPs often observe a discrepancy between the nature of the symptom and the patient’s presentation (e.g. catastrophizing). Also FPs believe that these patients predominantly hope to find reassurance in all kinds of additional examinations and referrals. These factors often negatively affect the empathy FPs feel and therewith clues for MUPS. FPs also mentioned that they know families in which all family members tend to worry about symptoms and have similar coping strategies. When the FP meets a member of such a family, they often consider MUPS early-on.

FPs indicated that they find it difficult to use anxiety as a signifier for MUPS in patients who have had a serious somatic disease (e.g. chest pain in patients with a myocardial infarction in their history). The extent of the patient’s anxiety may currently have no ground, but FPs consider it to be understandable and therefore justifiable which makes FPs also more inclined to keep searching for somatic explanations.

### The unhappy MUPS patient

FPs stated that there are several patients with MUPS with a low mood but that they find it difficult to distinguish between a primarily depressive disorder, where patients also express physical symptoms, and patients with primarily MUPS that are accompanied by a low mood caused by their symptoms. However, it was clear to them that a psychiatric diagnosis of a depressive disorder rules out MUPS as a primary diagnosis. According to FPs, patients with MUPS with a low mood present themselves as unhappy and sometimes apathetic during consultations. However, FPs noticed that the mood of these patients is not as seriously disturbed as in depressed patients and fluctuates over the recurrent consultations, where they mostly present physical symptoms as their main problem. FPs indicated that sometimes it takes time and several consultations to distinguish between MUPS and a depressive disorder.

### The passive MUPS patient

According to the FPs, these patients feel that they have no control over their life and that events in daily life just happen to them. This feeling of having no control might be the result of a traumatic history such as childhood abuse.

FPs stated that these patients present themselves as helpless during consultations, show little capacity for introspection and externalize their problems, which can lead to a difficulty in accepting that social or psychological problems might play a role in their complaints. Several FPs described their behaviour as ‘trash bin emptying’, where patients lean backwards after having spilled their complaints and wait until the FP offers the solution. This often evokes negative feelings, such as irritation or resistance, in FPs.

### The distressed MUPS patient

The FPs believe that they recognize the distressed patient with MUPS quickly. The majority of distressed patients with MUPS verbalizes their symptoms well and are willing and capable to attribute their symptoms to circumstances or psychosocial issues. In some cases, patients lack insight into these psychosocial issues and therefore are more fixed on the physical symptoms, which could lead to conflicts during consultations in which the FP tries to discuss non-physiological causes or influences.

Broadly FPs named three underlying causes for distress: 1) a phase in life where several things come together (e.g. relational/work problems). In this case MUPS are more often acute than chronic, but can be recurrent. FPs consider this combination of life events and symptoms as a variant of normal life; 2) perfectionism and burn-out, where patients are often highly educated and successful; 3) poor external life situations (e.g. financial strain/long and heavy working hours and migrants) and no social support system; a general vulnerability.

### The puzzling MUPS patient

Most FPs believe that there are almost always circumstances, habits, coping strategies or personality traits that sustain or (partially) cause MUPS. However, according to FPs there is a small group of patients where the FP cannot rely on existing explanatory models to substantiate MUPS as a working hypothesis and therefore has no clue why the patient experiences symptoms. Some FPs suspect a physiological cause that science has not discovered yet.

FPs reported that patients in this subgroup tell a clear story about very specific complaints and FPs indicated that they are more inclined to refer them for additional diagnostics or to a specialist in order to search together for an explanation.

The member check revealed that our findings were consistent between the FPs who participated in our study and FPs who did not participate in our study.

## Discussion

In our study, we found that FPs not only recognize patients with MUPS by using more or less objective data such as frequent and long consultations, but that subjective feelings such as irritation and resistance contribute to recognition as well. Recognition is also influenced by the patient’s ability to tell his/her story, doctor characteristics such as years of life and working experience, the doctor-patient relationship and the knowledge the doctor has of his/her patients’ history and context. In these characteristics we recognize the values of family medicine. Five subgroups of patients according to FPs could be distinguished from the focus group discussions data: the anxious, the unhappy, the passive, the distressed and the puzzling patient with MUPS. The last subgroup, the puzzling patient, is somewhat different than the other four and does not include the more general characteristics of patients with MUPS according to FPs.

### Strengths and limitations

Our study has several strong points. As far as we know, our study is the first that addresses recognition of patients with MUPS and distinct subgroups based on perceptions and experience of FPs with this methodology. By starting with the analysis of previously conducted focus group discussions, we laid a strong foundation for the new ones. We validated our results with a member check among participating and non-participating FPs to strengthen our findings. As we assembled and took into account a wide variety of opinions by using a purposive sampling strategy, our results address and illuminate many aspects of the recognition of patients with MUPS. Finally, researchers from several disciplines (FPs, a psychologist and a methodologist) worked together in this study, thereby providing insights from different fields.

However, our findings should be interpreted in the light of several limitations. First, the focus groups gave insight in the thoughts of FPs, but the actual clinical practice could be different from what they reported during the focus groups. Besides, as the focus group discussions were audiotaped, we did not take into account non-verbal signals. Our focus groups consisted of FPs, doctors from other somatic specialties could come up with different subgroups. Finally, we do not know if patients recognize themselves in the emerged subgroups. It is possible that they have other perceptions about their symptoms which could lead to difficulties during consultations or lack of adherence to treatment plans.

### Comparison with existing literature

Our study results can be compared with those of some other studies. Our findings regarding the recognition of MUPS correspond with a focus group study from Schou Hansen et al [17]. Although their study had a broader focus (i.e. employment of the MUPS definition and MUPS management), they also found that the process of recognition is shaped during the consultation. Mik-Meyer et al showed that FPs not only use traditional biomedical diagnostic tools but also rely on their own opinions and feelings and evaluations of a patient’s context and circumstances, comparable to what we found [18]. Two studies support our findings that a continuous doctor-patient relationship with knowledge of the patient and his/her context facilitates recognition [19, 20].

We found one study regarding different subgroups of undifferentiated MUPS. Rosmalen et al performed latent class analysis and found two classes, in which the number of symptoms was distinctive [21].

There are several studies that specified subgroups within the specific functional MUPS syndromes. Van Koulil et al found two subgroups of FM patients: patients with pain avoidance and patients with pain persistence, while Turk et al found three groups: the dysfunctional group, the interpersonally distressed group and the adaptive copers group [7, 8]. Both authors concluded that different groups of patients benefited from different treatments. The characteristics of these subgroups such as passivity and distress are somewhat similar to our findings. Cella et al found with their latent class analysis that one class of patients with CFS, with a predominance of anxiety and a symptom focus, predicted a poor response to CBT [10]. Finally, Viniol et al found three clusters of patients with chronic low back pain (CLBP) with a cluster analysis that could influence further treatment [22].

There are some studies that underpin the relationship between MUPS and the characteristics that were predominant in our subgroups. Several studies confirm the comorbidity between anxiety and MUPS and depressive feelings and MUPS [23, 24]. Burton et al found that FPs mostly saw worry as a trait coinciding with MUPS rather than as a symptom of an anxiety disorder and low mood as a response to circumstances that could also be a symptom of a depressive disorder [23]. Van Gils et al showed that an increase in stress precedes an increase in physical symptoms in some individuals, but is not an universal predictor of MUPS [25]. Our findings point in the same direction where FPs considered distress to be a pronounced and sustaining factor. Kempke et al found that perfectionism was related to severity of fatigue and low mood in patients with CFS [26, 27].

Finally, the characteristics of our subgroups are not exclusively linked to MUPS. Patients with chronic somatic diseases, such as chronic lung disorders and diabetes, may also suffer from anxiety and depressive symptoms [28–30]. However, FPs use these mental symptoms as a diagnostic tool for the recognition of MUPS, in contrast to chronic somatic diseases.

Bombardier showed that different psychological types found among CLBP patients are also common among patients with a wider range of chronic medical conditions. And consequently, effective elements of treatment as provided for those conditions could also be used in MUPS management [31].

### Implications for clinical practice and future research

With our study we gained more in depth insight into the process of recognition of MUPS by FPs. By being alert on signifiers of MUPS during consultations FPs may more quickly recognize MUPS thus allowing them to pay extra attention to the doctor-patient relationship early on. A good and continuous doctor-patient relationship consisting of positive communication, support, empathy is imperative for good MUPS management and leads to better health outcomes. Finally, by quickly recognizing MUPS and adequate treatment, chronicity of MUPS might be prevented.

The subgroups need to be tested and validated. Therefore we suggest both qualitative and quantitative observational studies of consultations between patients and FPs. In these studies, the following research questions should be addressed: what is the prevalence of each subgroup, how do the subgroups overlap, what is the course and outcome per subgroup and which treatments are adequate and acceptable and do treatment effects differ across subgroups? When the subgroups are confirmed and validated, they might be helpful for guiding more personalized treatment. Furthermore, when our findings are validated, they could contribute to the current discussions about the classification and definition of MUPS in family medicine and could have added value for the MUPS guidelines [15, 32]. Finally, we believe a future qualitative study should include the perspective of the patient regarding recognition by FPs. Tschudi-Madsen et al showed that patients frequently consider that they may suffer from MUPS [33]. In the proposed future study patients should be asked what their perceptions are regarding these subgroups and whether they believe these subgroups will be helpful for targeting treatment.

## Conclusion

With our study we gained more insight into the complex process of recognition of patients with MUPS by FPs. Recognition is strongly connected with the values of family medicine: the doctor-patient relationship, knowledge of a patient’s context and continuous care. We were able to partly unravel the heterogeneity among MUPS patients as five subgroups could be distinguished according to FPs, based on certain explicit or predominant characteristics. It is possible that all patients with MUPS require the same basic treatment, with a focus on good doctor-patient communication and a good relationship, but that the subsequent treatment steps require a different work-up, as different patients may have different needs. Personalizing treatment in this way could improve quality of care. However, further research, that should also include the patient’s perspective, has to be conducted to confirm and validate the different subgroups.

### Ethical approval and consent to participate

The medical ethics committee VUmc approved the study (reference number 2015.216). All FPs provided written informed consent to participate, for the usage of the data and for publication of this manuscript.

### Consent for publication

Not applicable.

### Availability of data and materials

The databases and materials will be made available on request.

## Abbreviations

CFS: chronic fatigue syndrome
CLPB: chronic low back pain
EMRs: electronic medical records
FM: fibromyalgia
FPs: family physicians
MUPS: medically unexplained physical symptoms
